# An open-label, randomized, single intravenous dosing study to investigate the effect of fixed-dose combinations of tenofovir/lamivudine or atazanavir/ritonavir on the pharmacokinetics of remdesivir in Ugandan healthy volunteers (RemTLAR)

**DOI:** 10.1186/s13063-021-05752-1

**Published:** 2021-11-23

**Authors:** Stephen I. Walimbwa, Julian P. Kaboggoza, Catriona Waitt, Pauline Byakika-Kibwika, Antonio D’Avolio, Mohammed Lamorde

**Affiliations:** 1 Infectious Diseases Institute, Makerere University College of Health Sciences, Kampala, Uganda; 2 Department of Molecular and Clinical Pharmacology, University of Liverpool, Liverpool, UK; 3 Department of Medicine, Makerere University, Kampala, Uganda; 4 Laboratory of Clinical Pharmacology and Pharmacogenetics, Amedeo di Savoia Hospital, Department of Medical Sciences, University of Turin, Turin, Italy; 5 CoQua Lab, Turin, Italy

**Keywords:** Remdesivir, COVID-19, Ebola, HIV, Atazanavir, Ritonavir, Tenofovir, Lamivudine, Drug-drug interactions

## Abstract

**Background:**

Remdesivir is a novel broad-spectrum antiviral therapeutic with activity against several viruses that cause emerging infectious diseases. The purpose of this study is to explore how commonly utilized antiretroviral therapy (tenofovir disoproxil fumarate/lamivudine [TDF/3TC] and atazanavir/ritonavir [ATV/r]) influence plasma and intracellular concentrations of remdesivir.

**Methods:**

This is an open-label, randomized, fixed sequence single intravenous dosing study to assess pharmacokinetic interactions between remdesivir and TDF/3TC (Study A, crossover design) or TDF/3TC plus ATV/r (Study B). Healthy volunteers satisfying study entry criteria will be enrolled in the study and randomized to either Study A; *N*=16 (Sequence 1 or Sequence 2) or Study B; *N*=8. Participants will receive standard adult doses of antiretroviral therapy for 7 days and a single 200mg remdesivir infusion administered over 60 min. Pharmacokinetic blood sampling will be performed relative to the start of remdesivir infusion; predose (before the start of remdesivir infusion) and 30 min after the start of remdesivir infusion. Additional blood samples will be taken at 2, 4, 6, 12, and 24 h after the end of remdesivir infusion.

**Discussion:**

This study will characterize the pharmacokinetics of remdesivir from a typical African population in whom clinical use is anticipated. Furthermore, this study will deliver pharmacokinetic datasets for remdesivir drug concentrations and demographic characteristics which could support pharmacometric approaches for simulation of remdesivir treatment regimens in patients concurrently using tenofovir/lamivudine and/or atazanavir/ritonavir.

**Trial registration:**

ClinicalTrials.gov NCT04385719. Registered 13 May 2020.

## Administrative information

Note: the numbers in curly brackets in this protocol refer to SPIRIT checklist item numbers. The order of the items has been modified to group similar items (see http://www.equator-network.org/reporting-guidelines/spirit-2013-statement-defining-standard-protocol-items-for-clinical-trials/).

**Table:** 

Title {1}	An open-label, randomized, single intravenous dosing study to investigate the effect of fixed-dose combinations of tenofovir/lamivudine or atazanavir/ritonavir on the pharmacokinetics of remdesivir in Ugandan healthy volunteers (RemTLAR).
Trial registration {2a and 2b}.	ClinicalTrials.gov, ID:NCT04385719
Protocol version {3}	Protocol Version 3.0, 21 September 2020
Funding {4}	This study is part of leveraging capacity for early phase clinical trials for filoviruses in Uganda (CAPA-CT 2) consortium funded by the European and Developing Countries Clinical Trials Partnership (RIA2018EF-2083).
Author details {5a}	Stephen I Walimbwa^1^, Julian P Kaboggoza^1^, Catriona Waitt^1,2^, Pauline Byakika-Kibwika^1,3^ , Antonio D’Avolio^4,5^, Mohammed Lamorde^1^ 1. Infectious Diseases Institute, Makerere University College of Health Sciences, Kampala, Uganda 2. Department of Molecular and Clinical Pharmacology, University of Liverpool, Liverpool, UK 3. Department of Medicine, Makerere University, Kampala, Uganda 4. Laboratory of Clinical Pharmacology and Pharmacogenetics, Amedeo di Savoia Hospital, Department of Medical Sciences, University of Turin, Turin, Italy 5. CoQua Lab, Turin, Italy
Name and contact information for the trial sponsor {5b}	Dr. Andrew Kambugu Infectious Diseases Institute, Makerere University College of Health Sciences. P.O. Box 22418, Kampala, Uganda. Telephone: +256 312 211435 Ext. 435 akambugu@idi.co.ug
Role of sponsor {5c}	All originating RemTLAR data is the property of the sponsor. The funder has no role in the design of the study and collection, analysis, and interpretation of data and in writing the manuscript.

## Introduction

### Background and rationale {6a}

In sub-Saharan Africa, there is a significant geographic overlap between chronic infectious diseases like HIV and emerging infectious diseases like coronavirus disease 2019 (COVID-19) and outbreaks of filoviruses. Consequently, drug-drug interactions between highly efficacious antiretroviral therapy (ART) given to people living with HIV and experimental therapies against COVID-19 and filoviruses may occur.

Remdesivir (GS-5734) is a nucleoside analog with in vitro activity against filoviruses Zaire, Sudan, and Bundibugyo Ebola viruses and Marburg virus, in addition to arenaviruses and pathogenic coronaviruses (middle east respiratory syndrome (MERS) and severe acute respiratory syndrome coronavirus (SARS Cov)) and paramyxoviruses (Nipah and Hendra) [1, 2]. Remdesivir’s broad-spectrum antiviral activity improves the current armamentarium of therapies used during outbreaks of international concern.

In December 2019, a novel coronavirus outbreak caused by the SARS-COV-2 virus emerged in China and caused a global public health emergency of international concern that was subsequently declared a pandemic. Remdesivir was identified as having in vitro activity against SARS-COV-2 and fast-tracked as a possible investigational therapeutic for the management of COVID-19 [1, 3]. In adults and pediatric patients hospitalized with COVID-19, remdesivir received approval and Emergency Use Authorization (EUA) from the US FDA [4]. Whereas the optimal treatment duration and efficacy in patients with COVID-19 is yet to be confirmed, remdesivir is administered for a total 5–10 days or until hospital discharge. In adult patients, the recommended dose is 200mg on day 1, followed by once-daily maintenance doses of 100mg from day 2 [5]. For pediatric patients less than 12 years of age weighing 3.5–<40kg, only the lyophilized formulation can be used at 5mg/kg on day 1, followed by once daily maintenance doses of 2.5 mg/kg from day 2 [6].

In a rhesus monkey model of Ebola virus disease (EVD), once daily administration of remdesivir at a dose of 10mg/kg resulted in profound suppression of Ebola virus (EBOV) replication and protected 100% of infected animals against lethal disease, ameliorating clinical disease signs and pathophysiological markers [7–9]. However, an interim analysis of the PALM clinical trial that was comparing four therapeutics for the Zaire Ebolavirus outbreak (mab114, REGN-EB3, remdesivir, and ZMapp) decided to stop the study and randomize all remaining patients to either one of two monoclonal antibody-based therapies mAb114 or REGN-EB3. Among all patients (*n*=499) who took the drugs, mortality rates were REGN-EB3, 29%; mAb-114, 34%; ZMapp, 49% and remdesivir, 53% [10]. Although remdesivir performed poorly compared to other products, it remains relevant as it is the only investigational therapeutic in development for Sudan and Bundibugyo Ebola viruses for its potential to reach sanctuary sites and possible use in the future as part of combination strategies for EVD with monoclonal antibodies [11]. Furthermore, the use of remdesivir a therapeutic with pan-filovirus potential is desirable for a simplified and sustainable medical countermeasures strategy for viral hemorrhagic fevers.

First-line ART regimens in Uganda and in many other sub-Saharan countries contain a nucleotide reverse transcriptase inhibitor (NRTI) tenofovir disoproxil fumarate (TDF) used in combination with lamivudine (3TC), a nucleoside reverse transcriptase inhibitor (NRTI), and a third drug to complete the regimen. Second- and third-line ART treatment options often contain ritonavir-boosted protease inhibitors (PIs) like atazanavir/ritonavir (ATV/r) which have a high potential for drug-drug interactions [12, 13]. Following oral administration, TDF is rapidly hydrolyzed to tenofovir and formaldehyde without CYP involvement. Tenofovir then undergoes intracellular phosphorylation to its active metabolite tenofovir diphosphate which inhibits the activity of HIV-1 reverse transcriptase by competing with the natural substrate, deoxyadenosine 5′-triphosphate, and by DNA chain termination [14]. Lamivudine is phosphorylated intracellularly to its active metabolite lamivudine 5′-triphosphate and is predominately cleared unchanged by renal excretion and transported by MRP4 and MRP8. Atazanavir is metabolized primarily by hepatic CYP enzymes 3A4/3A5 and transported by P-glycoprotein (P-gp) and MRP1. Ritonavir is metabolized by CYP 3A4 and transported by P-gp, MRPs, and BCRP. Clinically significant interactions involving NRTIs and PIs and have been reported due to agonistic/antagonistic effects on CYP enzymes and transporters with drugs commonly used across sub-Saharan Africa [15, 16].

Remdesivir is a substrate of cytochrome P 450 (CYP) enzymes 2C8, 2D6, and 3A4 which are prone to induction and inhibition. Remdesivir is metabolized to its pharmacologically active nucleoside triphosphate within multiple human cell types relevant for replication of a wide range of viruses. When co-administered with tenofovir disoproxil fumarate, the potential for drug-drug interactions might be exhibited by inhibition of multidrug resistance-associated protein 4 (MRP4) a transporter inhibited by remdesivir. Furthermore, tenofovir the pharmacologically active form of TDF and remdesivir are both adenine nucleoside analogs that undergo intracellular phosphorylation; co-administration of antiretrovirals may alter remdesivir intracellular concentrations or pharmacodynamics. Therefore, there is an urgent need to evaluate the drug-drug interactions between remdesivir and commonly used antiretroviral therapies.

### Objectives {7}

#### Primary objective

1. To assess the safety and tolerability of single intravenous doses of remdesivir in adult healthy volunteers
2. To evaluate the intracellular pharmacokinetics of single-dose intravenous remdesivir with or without co-administration of oral fixed-dose combination tenofovir/lamivudine with patients serving as their own controls

#### Secondary objective

1. To evaluate the difference in plasma and intracellular pharmacokinetics of intravenous remdesivir among healthy volunteers receiving tenofovir/lamivudine versus healthy volunteers receiving tenofovir/lamivudine plus atazanavir/ritonavir tablets.
2. To generate a population pharmacokinetic model to describe inter-individual variability in intracellular pharmacokinetics of remdesivir

### Exploratory objectives

1. To describe polymorphic variants of relevant kinases that activate tenofovir and explore possible consequences on remdesivir pharmacokinetics.

### Trial design {8} (Fig. 1)

This is an open label, randomized, fixed sequence single intravenous dosing study to assess pharmacokinetic interactions between remdesivir and TDF/3TC (Study A, crossover design) or TDF/3TC plus ATV/r (Study B). An essential design feature of this study is that the impact of perpetrators on the pharmacokinetics of remdesivir will be collected under target saturating conditions of the antiretrovirals.

**Fig. 1:**
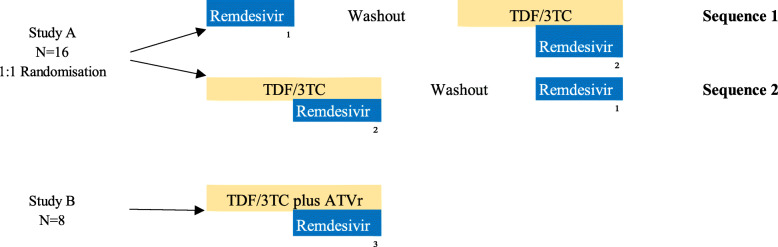
Study schema

## Methods: Participants, interventions, and outcomes

### Study setting {9}

All study visits and blood sampling will be conducted in a 2-bed phase II ward at Infectious Diseases Institute (IDI) Clinical Trials Unit (CTU) in Kampala, Uganda. The IDI outpatient clinic provides prevention, care, and treatment services for infectious diseases and antiretroviral therapies to over 8000 people living with HIV.

### Eligibility criteria {10}

Participants who meet all the following inclusion criteria are eligible for enrolment into the study:

### Inclusion criteria

1. Evidence of a personally signed and dated informed consent document indicating that the participant has been informed of all pertinent aspects of the study.

2. Participants who are willing and able to comply with scheduled visits, treatment plan, laboratory tests, and other study procedures.

3. Healthy men and women aged 18 to 55 years of age, and in good health as determined by past medical history, physical examination, vital signs, electrocardiogram, and laboratory tests at screening.

4. At screening, vital signs (systolic and diastolic blood pressure and pulse rate) will be assessed in the sitting position and again (when required) in the standing position. Sitting vital signs should be within the following ranges:

I. Axillary body temperature between 35.5 and 37.0°C
II. Systolic blood pressure, 90–139 mmHg
III. Diastolic blood pressure, 50–89 mmHg
IV. Pulse rate, 50–90 bpm.

5. Subjects must weigh at least 40 kg to participate in the study and must have a body mass index (BMI) within the range 18–30 kg/m^2^. BMI= body weight (kg)/[height (m)]^2^

6. HIV antibody negative at screening.

7. Women of childbearing potential must be willing to use a highly effective contraception method (e.g., IUD or hormonal contraceptive implant, complete abstinence (if genuinely followed)) or consistent use of a barrier method such as male or female condoms plus oral progestin contraceptives for the duration of the study. Non-surgically sterilized men must agree to abstain from sexual intercourse for the duration of the study or use condoms for contraception for the duration of the study.

8. Hemoglobin concentration equal or greater than 10 g/dL

Participants with any of the following will be excluded from the study:

### Exclusion criteria

1. Significant disease affecting cardiac, respiratory, gastrointestinal, or neurological symptoms which in the clinician’s medical judgment could be worsened by participating in this study or the presence of medical or surgical conditions which could prevent the subject from complying with study procedures.

2. Serum alanine transaminase (ALT) levels above 2x upper limit of normal (ULN) or total bilirubin > 1.3x ULN

3. Hepatitis B surface antigen positive

4. Serum creatinine levels above 1.5x upper limit of normal

5. Evidence of QT prolongation on electrocardiogram (ECG) QTc (rate adjusted QT interval) > 450ms (men) or 460ms (women)

6. Pregnant women or female subjects who are unwilling to use a suitable contraceptive method for the duration of the study (IUD or contraceptive implant)

7. Likely to be poorly adherent based on clinician’s medical judgement

8. Known to be current injection drug user

### Who will take informed consent? {26a}

Consent will be taken by members of the study team who have been delegated the responsibility of taking informed consent by the Principal Investigator. Informed consent will be obtained and documented prior to the participant undergoing study procedures. The informed consent document will comply with GCP and local regulatory guidelines.

Informed consent will be obtained in either English or Luganda. The investigator will retain the original of each participant’s signed consent document. For participants unable to read and/or write, an independent witness will be involved in the consent process.

All parties will ensure protection of participant personal data and will not include participant names on any forms, reports, publications, or in any other disclosures, except where required by and legal requirements.

The informed consent document used in this study, and any changes made during the course of the study, will be prospectively approved by the Research Ethics Committee/Institutional Review Board (REC/IRB).

Participants are free to withdraw at any time from the study without providing a reason. Data and samples collected up to the point of withdrawal of consent may be used unless the participant specifically withdraws consent for use of these data and samples.

### Additional consent provisions for collection and use of participant data and biological specimens {26b}

Consent will be sought for storage of biological samples and for exploratory pharmacogenomic research. Exploratory pharmacogenetic research which may include genome-wide association studies (GWAS) is optional and does not exclude participation if declined.

### Interventions

#### Explanation for the choice of comparators {6b}

##### Rationale for tenofovir/lamivudine and anticipated interactions with remdesivir

Although the parenteral route of administration for remdesivir minimizes the potential impact from gastro-intestinal transporters, there are no clinical data on the potential interaction between antiretroviral NRTIs and remdesivir. It is not known whether a dose modification is needed when tenofovir/lamivudine and remdesivir are co-administered. A lead-in (pre-treatment) phase of seven once daily doses of TDF/3TC has been selected to maximize the potential interaction of TDF/3TC at steady state.

Potential for genetic variation exists among kinases (adenylate kinase 2 (AK2) creatine kinase, muscle (CKM) pyruvate kinase, muscle (PKM) and pyruvate kinase, liver, and red blood cell (PKLR)) responsible for intracellular phosphorylation of tenofovir [17]. Known polymorphisms considered likely to alter intracellular exposure of tenofovir will be described [18].

##### Rationale for atazanavir/ritonavir and anticipated interactions with remdesivir

Remdesivir is a substrate of both CYP3A4 (in vitro) and P-gp and is predominately metabolized by hydrolase activity. Ritonavir (RTV) is a well-established strong dual inhibitor of CYP3A4 and P-gp. Although potential for clinically significant drug interactions is considered low, the effect of simultaneous inhibition of both CYP3A4 and P-gp by ritonavir (RTV) could result in a drug-drug interaction [19, 20]. Exposures of prototype P-gp substrates such as digoxin and fexofenadine are increased with single and multiple dosing with ritonavir [21].

After short lead-in (pre-treatment), very rapid and potent inhibition of both hepatic and intestinal CYP3A4 have been seen whose mechanism has been postulated to have both a reversible and time-dependent component [21, 22]. For this study, a pretreatment phase of seven once daily doses of ATV/r has been selected to maximize the potential interaction. Additionally, co-administration of tenofovir disoproxil fumarate (300 mg once daily) with atazanavir/ritonavir (300 mg/100 mg once daily) was studied in healthy volunteers. Tenofovir disoproxil fumarate area under concentration-time curve (AUC), maximum concentration (C_max_), and minimum concentration (C_min_) increased 37%, 34%, and 29%, respectively. The mechanism of interaction between atazanavir and tenofovir disoproxil fumarate is unknown.

#### Intervention description {11a}

Eligible participants will be randomized into Study A (Sequence 1 or Sequence 2) in a 1:1 ratio or Study B using a randomization schedule.

Intravenous infusions of remdesivir 200mg will be administered over 60 min (1 h) by the study personnel to all participants in accordance with the treatment schedule. Remdesivir infusion will be prepared by aseptic technique and the preparation administered immediately. Remdesivir for injection lyophilized powder will be reconstituted with 19ml of Sterile Water for Injection and further diluted with 0.9% sodium chloride infusion bag prior to administration. Care will be taken during admixture to prevent inadvertent microbial contamination.

Standard adult doses of generic TDF/3TC (300mg/300mg) (Study A) or TDF/3TC (300mg/300mg) plus ATV/r (300mg/100mg) (Study B) will be administered under direct observation (DOT) on the first day and thereafter once daily self-administered by the study participants prior to intensive pharmacokinetic visits during which observed dosing will be implemented.

Adherence to pre-treatment schedules will be assessed by pill counts at study visits by direct questioning of dosing schedules and missed doses, self-report, and by measurement of drug concentrations in the urine and blood using high performance liquid chromatography (HPLC) and liquid chromatography-tandem mass spectrometry (LC-MS/MS).

At each study visit, history of adverse events (AEs) will be collected, and vital signs will be monitored. Complete physical examinations will be performed at the screening and follow-up visits if clinically indicated. Mandatory safety laboratory investigations and follow-up procedures are specified in the visit schedule.

#### Criteria for discontinuing or modifying allocated interventions {11b}

Discontinuation of study treatment for a participant occurs when remdesivir or pre-treatment with TDF/3TC or ATV/r is stopped earlier than the protocol planned duration. Discontinuation of study treatment can be decided by either the participant or the investigator.

Study treatment must be discontinued under the following circumstances:

- Participant decision—participants may choose to discontinue study treatment for any reason at any time.
- The investigator believes that continuation would negatively impact the safety of the subject
- Any protocol deviation that results in a significant risk to the subject’s safety.
- Common Terminology Criteria for Adverse Effects (CTCAE) Grade 3 or higher adverse event unless it can be conclusively shown that AE is not related to study treatment.
- Use of prohibited treatment
- Moderate to severe hypersensitivity reaction to study drugs
- Any laboratory abnormalities that in the judgment of the investigator, taking into consideration the subject’s overall status, prevents the subject from continuing participation in the study.
- Pregnancy

Discontinuation of study treatment will be at the discretion of the Investigator, under the following circumstances:

- Serious adverse event that is considered to be related to study drugs
- Two or more AEs showing similar symptoms or having same diagnosis graded CTCAE 3 and being considered related to the administration of study drugs

If discontinuation of study treatment occurs, the investigator must determine the primary reason for the subject’s premature discontinuation of study treatment and record this information in the subjects case report forms (CRFs).

Participants who discontinue study treatment or who decide they do not wish to participate in the study further should NOT be considered withdrawn from the study UNLESS they withdraw their consent. Where possible, they should return to the site and complete the End of Study visit. If they fail to return for these assessments for unknown reasons, every effort (e.g., telephone, home visit, letter) should be made to contact them.

#### Strategies to improve adherence to interventions {11c}

Initial pre-treatment doses of tenofovir/lamivudine and atazanavir/ritonavir will be administered under Direct Observation/Directly Observed Therapy (DOT) on the first day and thereafter once daily self-administered by the study participants prior to intensive pharmacokinetic visits during which observed dosing will be implemented.

Adherence to pre-treatment schedules will be assessed by pill counts at study visits by direct questioning of dosing schedules and missed doses, telephone visits, self-report, and therapeutic drug monitoring (TDM) of drug concentrations in urine and blood using HPLC and LC-MS/MS.

#### Relevant concomitant care permitted or prohibited during the trial {11d}

Except for medication which may be required to treat adverse events, no medication other than study drugs will be allowed from the first dosing until all of the end of study evaluations have been conducted. Medicines that are specifically contraindicated with remdesivir or any of the study drugs should not be used. The current remdesivir investigators brochure and summary of product characteristics for tenofovir/lamivudine and atazanavir/ritonavir will be referred to for contraindicated medications. Both prior and concomitant medication prescribed during the course of the study will be documented.

#### Provisions for post-trial care {30}

All participants with post-recruitment illness will be monitored until symptoms resolve, laboratory changes return to baseline or until there is a satisfactory explanation for the changes observed. Patients will receive tertiary level medical care including admission at the National Referral Hospital (Mulago/Kiruddu Hospital), and patients will be managed in accordance with Uganda national treatment guidelines.

#### Outcomes {12}

##### Endpoints to primary objectives

1. Adverse events, physical exam findings, vital signs (blood pressure, body temperature, respiratory rate), ECG findings (QTc, PR, QRS intervals), safety laboratory assessments, and SAEs
2. Single pharmacokinetic parameters in peripheral blood mononuclear cells (PBMCs): maximum intracellular concentration [Cmax], time to maximum concentration [Tmax], elimination rate constant, area under the concentration-time curve [AUCinf, AUClast], and half-life (t½) of remdesivir

##### Endpoint to secondary objective

A population pharmacokinetic model of remdesivir

##### Endpoints to exploratory objectives

Pharmacogenomic characterization of metabolic enzymes involved in remdesivir metabolism.

##### Participant timeline {13} (Tables 1, 2 and 3)

Study A

**Table 1:** Sequence 1

Protocol activity	Screening*	Rando**	Day 1	Day 2	Days 3–7	Day 8	Days 9–13	Day 14	Day 15	Day 18	Day 22	Day 29 EOS
Informed consent	**X**											
Enrolment		**X**										
Remdesivir			**X**					**X**				
TDF/3TC						**X**	**X**	**X**				
Rich PK visit			**X**					**X**				
Single PK sample				**X**					**X**			
Washout period												
Clinical review	**X**		**X**		**X**^**T**^	**X**	**X**^**T**^	**X**		**X**^**T**^		**X**
Vital signs	**X**		**X**					**X**				**X**
HBV serology	**X**											
HIV serology	**X**											
Malaria RDT	**X**											
Safety bloods	**X**			**X**		**X**		**X**	**X**		**X**	
Urinalysis	**X**		**X**					**X**				
Pregnancy test	**X**		**X**			**X**						
ECG	**X**		**X**					**X**				
Pharmacogenomic sample						**X**						

*14-day screening period, **At least 3 days prior to drug administration, *X*^*T*^ telephone visit, *RDT* rapid diagnostic test, *Rando* randomization

Study A

**Table 2:** Sequence 2

Protocol activity	Screening*	Rando**	Day 1	Days 2–6	Day 7	Day 8	Days 9–14	Day 15	Day 16	Day 18	Day 22	Day 29 EOS
Informed consent	**X**											
Enrolment		**X**										
Remdesivir					**X**			**X**				
TDF/3TC			**X**	**X**	**X**							
Rich PK visit					**X**			**X**				
Single PK sample						**X**			**X**			
Washout period												
Clinical review	**X**		**X**	**X**^**T**^			**X**^**T**^	**X**		**X**^**T**^		**X**
Vital signs	**X**		**X**					**X**				**X**
HBV serology	**X**											
HIV serology	**X**											
Malaria RDT	**X**											
Safety bloods	**X**				**X**	**X**		**X**	**X**		**X**	
Urinalysis	**X**				**X**							
Pregnancy test	**X**		**X**					**X**				
ECG	**X**				**X**			**X**				
Pharmacogenomic sample						**X**						

*14-day screening period, **At least 3 days prior to drug administration, *X*^*T*^ telephone visit, *RDT* rapid diagnostic test, *Rando* randomization

**Table 3:** Study B

Protocol activity	Screening*	Rando**	Day 1	Days 2–6	Day 7	Day 8	Day 11	Day 15	Day 22 EOS
Informed consent	**X**								
Enrolment		**X**							
Remdesivir					**X**				
TDF/3TC plus ATVr			**X**	**X**	**X**				
Rich PK visit					**X**				
Single PK sample						**X**			
Washout period									
Clinical review	**X**		**X**	**X**^**T**^			**X**^**T**^		**X**
Vital signs	**X**		**X**						**X**
HBV serology	**X**								
HIV serology	**X**								
Malaria RDT	**X**								
Safety bloods	**X**				**X**	**X**		**X**	
Urinalysis	**X**				**X**				
Pregnancy test	**X**				**X**				
ECG	**X**				**X**				
Pharmacogenomic sample						**X**			

*14-day screening period, **At least 3 days prior to drug administration, *X*^*T*^ telephone visit, *RDT* rapid diagnostic test, *Rando* randomization

### Sample size {14}

The primary objective will evaluate drug exposure of remdesivir in a uniform population with subjects acting as their own controls. A formal power calculation based on differences in exposure between two different groups is therefore not applicable in this situation. Intensive pharmacokinetic studies aim to estimate the pharmacokinetic parameters with precision, and therefore for studies with an intensive sampling schedule, as in this case, a sample size of 24 participants is considered sufficient.

### Recruitment {15}

Healthy volunteer participants will be identified and recruited from communities that are within close proximity of the IDI CTU. Input from the IDI Community Advisory Board (CAB) whose members are solicited from within communities where research is undertaken will be sought when sensitizing the community on the objectives of the trial.

IRB approved study flyers will be displayed on notice boards and other easily accessible locations. Administrative clearance will be obtained from responsible authorities to display study flyers within the community. Contact information including the physical address of the clinical team will be provided on the flyers. An informed consent form will be provided to potential participants who contact the clinical team and wish to get information regarding the study. Potential participants will be given adequate time to review the informed consent form and an opportunity to ask any questions about the study.

### Assignment of interventions: allocation

#### Sequence generation {16a}

Eligible participants will be assigned to study groups in accordance with a computer-generated randomization schedule. The randomization schedule will be generated using permuted block randomisation by the study statistician.

#### Concealment mechanism {16b}

Enrolled participants will be allocated sequentially to treatment groups using concealed envelopes. Only the study statistician will have access to the treatment allocation concealment log (TCL). Following the TCL, a set of sequentially numbered, opaque, and sealed envelopes for allocation concealment and treatment allocation will be prepared. Sealed and tamper-evident treatment allocation envelopes (TAE) will be prepared by the study statistician. One envelope will be created for each particular trial subject. Each envelope will have the study identification, e.g., the subject number to identify the particular subject recruited. The envelopes will be secured in a lockable drawer kept by the study pharmacist. Each envelope will contain a piece of white paper printed clearly with the subject number and the allocation given either as “Study A Sequence 1,” “Study A Sequence 2,” or “Study B.” When a particular trial subject has signed the written consent form and is eligible for randomization, the study clinician will assign a subject number chronologically to the subject. The chronological process will be maintained to make sure that the allocation is not subverted. Each allocation code will be verified with the central record kept with the study statistician.

#### Implementation {16c}

Confirmation of eligibility and enrolment of participants into the trial will be assigned to a medical practitioner. Enrolled participants will be allocated to the treatment arms by the study statistician who will maintain the computer-generated sequence and treatment allocation concealment log.

#### Assignment of interventions: blinding

##### Who will be blinded {17a}

Not applicable

##### Procedure for unblinding if needed {17b}

Not applicable

#### Data collection and management

##### Plans for assessment and collection of outcomes {18a}

Copies of the data collection tools are available upon request from the corresponding author.

##### Plans to promote participant retention and complete follow-up {18b}

Participants may voluntarily withdraw from the study for any reason at any time. They may be considered withdrawn if they state an intention to withdraw, fail to return for visits, or become lost to follow-up for any other reason. If a participant withdrawal occurs for any reason, the Investigator must make every effort to determine the primary reason for a subject’s withdrawal from the study and record this information in the CRF.

For participants who are lost to follow-up (i.e., those participants whose status is unclear because they fail to appear for study visits without stating an intention to withdraw), the study team will document in source documents steps taken to contact the participant, e.g., telephone calls, short message service (SMS), home visits, and letters/emails.

Participants who are withdrawn from the study for reasons other than safety may be replaced by an equal number of newly enrolled subjects.

Any participant who is discontinued for any of the above reasons and does not withdraw consent should return to the site to complete their end of study assessments.

If a participant withdraws from the study, and also withdraws consent for disclosure of future information, no further evaluations will be performed, and no additional data will be collected. The sponsor may retain and continue to use any data collected before such withdrawal of consent.

##### Data management {19}

Data will be entered into a study specific electronic database by designated staff on a regular basis from completed case report forms (CRFs) and stored securely on a server at Infectious Diseases Institute. Study records, including the identity of all participating participants, all original signed informed consent documents, copies of all CRFs, safety reporting forms, source documents, and detailed records of treatment disposition, and adequate documentation of relevant correspondence (e.g., letters, meeting minutes, telephone calls reports) will be kept securely in a locked cabinet with access granted only to authorized study staff. Upon study competition, study records will be transferred and stored at IDI’s secure archival site for at least 20 years in accordance with Uganda National Drug Authority (NDA) guidance.

##### Confidentiality {27}

Clinical data will be entered into a study specific electronic database by designated staff on a regular basis from completed Case Record Forms (CRFs). Access to the database will be given to authorized personnel only (members of the immediate study team), and a log of authorized personnel will be stored in the trial master file. Case report forms and trial documents will be kept in locked cabinets. No participant identifying information will be disclosed in any publication or at any conference activities arising from the study.

##### Plans for collection, laboratory evaluation, and storage of biological specimens for genetic or molecular analysis in this trial/future use {33}

Th whole blood will be obtained and collected into vacutainers containing anticoagulant or cell preparation tubes and delivered within 30 min to the laboratory. Samples will be centrifuged to separate the plasma and PBMCs. To inactivate the esterase responsible for remdesivir degradation, plasma samples will be heat inactivated at 58^o^C for 35 min and aliquoted into cryovials. The plasma and PBMC cryovials will be stored at −80°C until analysis. The whole blood or nucleate cell aliquots will be analyzed for DNA biomarkers (CYP gene alleles) related to the effect of treatment. Detailed descriptions of the assays will be included in a bioanalytical data report.

### Statistical methods

#### Statistical methods for primary and secondary outcomes {20a}

The study aims to evaluate the safety, tolerability, and pharmacokinetics of single-dose remdesivir in healthy volunteers. The number and percentage of adverse events will be tabulated by body system. All safety parameters will be listed by cohort, subject, and visit/time and summarized by treatment and visit/time. No formal statistical hypotheses of the safety or tolerability are to be tested.

Pharmacokinetic parameters in the plasma and PBMCs including the area under the concentration-time curve to the last measurable time point (AUC0-t), terminal elimination half-life (t1/2), maximum concentration (Cmax), and time to Cmax (Tmax) will be estimated using non-compartmental analysis (WinNonlin, Phoenix, version 6.1 or higher, Pharsight, Mountain View, CA). Remdesivir pharmacokinetic parameters with and without antiretroviral therapy will be compared by calculating geometric mean ratios and 90% confidence intervals. Coefficient of variation (CV=mean/SD*100) will be calculated at all timepoints and for the measured parameters. Only subjects with evaluable remdesivir pharmacokinetic parameters data will be included in the primary analysis.

Descriptive statistics (including mean, SD, CV% mean, geometric mean, CV%, median, minimum, and maximum) will be provided for all pharmacokinetic parameters of remdesivir.

#### Analysis of secondary endpoints

The pharmacokinetic data collected in the study will be interpreted using population pharmacokinetic modeling (pop-PK). A pop-PK model will be developed to describe remdesivir pharmacokinetics pooling the clinical trial pharmacokinetic data from each subject and each sequential treatment condition in a single analysis. Nonlinear mixed effects will be used to define remdesivir pharmacokinetic parameters and their relationships with covariates such as weight, age, and sex (fixed effects) and to determine interindividual (and potentially interoccasion) variability in parameters and residual variably (random effects). Simulations will be used to predict dosing for patients across the data range, for example, by weight, sex, and age groups. This will provide a quantitative evaluation of pharmacokinetic variability that could facilitate evaluation of potential dosing strategies for patients with special characteristics in this setting.

#### Interim analyses {21b}

A safety monitor will perform interim review of the trial’s safety data. The safety monitor will review safety data after the first five participants have been enrolled into the study and every 2 weeks thereafter. Further reviews may be convened at the discretion of the safety monitor, or upon request from the Principal Investigator if unexpected data emerge, or if the study stopping criteria is met.

#### Study stopping rules

Enrolment in the study will be placed on hold if any of the following occurs cumulatively across all of the cohorts:

- One or more treatment-emergent SAEs, except those that are clearly and incontrovertibly due to extraneous causes;
- At least two or more subjects experience a similar AE which is assessed as severe in intensity and is considered as potentially related to study drugs;
- The Principal Investigator and the safety monitor consider that the number and/or severity of AEs, abnormal safety monitoring tests, or abnormal laboratory findings justify putting the study on hold.

The study may resume if it is safe to proceed following a safety review of participant clinical and laboratory data.

#### Methods for additional analyses (e.g., subgroup analyses) {20b}

##### Analysis of exploratory endpoints

Stored samples will be used for optional genetic research which may include genome-wide association studies. As the numbers of participants enrolled in the study are too small to complete proper statistical analyses, additional data from other similar clinical trials, maybe combined, as appropriate, with those from other studies to enlarge the data set for analysis.

#### Methods in analysis to handle protocol non-adherence and any statistical methods to handle missing data {20c}

No imputation of missing data will be considered for safety and tolerability analysis.

#### Plans to give access to the full protocol, participant level-data, and statistical code {31c}

The full protocol, anonymous participant-level dataset, and statistical code will be made available upon request from the corresponding author.

#### Oversight and monitoring

##### Composition of the coordinating centre and trial steering committee {5d}

Dr. Mohammed Lamorde (principal investigator), Dr. Stephen I Walimbwa, Julian P Kaboggoza, Dr. Catriona Waitt, and Dr. Pauline Byakika-Kibwika will oversee the day to day activities of the trial.

##### Composition of the data monitoring committee, its role, and reporting structure {21a}

A safety monitor will make data safety and monitoring recommendations about the study. The safety monitor is a clinical trial research-experienced infectious diseases clinician without direct involvement in the study. The safety monitor will review safety data after the first five subjects have been enrolled into the study. Further reviews may be convened (at the discretion of the safety monitor, or upon request from the Principal investigator) if unexpected data emerge, or if the study stopping criteria is met. Any serious adverse events (SAEs) or Suspected Unexpected Serious Adverse Reaction (SUSARs) will also be reported to the safety monitor in a timely manner. A charter describing the functioning of the safety monitor has been developed.

##### Adverse event reporting and harms {22}

**Adverse events**

All observed or volunteered AEs regardless of treatment group or suspected causal relationship will be reported.

For all AEs, the investigators will pursue and obtain information adequate both to determine the outcome of the AE and to assess whether it meets the criteria for classification as an SAE requiring immediate notification. For all AEs, sufficient information will be obtained by the investigators to determine the causality of the AE. The investigators will assess causality. Follow-up by the investigators will be until the event or its sequelae resolve or stabilize at a level acceptable to the investigators.

During each study visit the study clinician will assess AEs which may have occurred since the previous visit. The investigators will generate and submit annual reports summarizing these adverse events.

**Serious adverse events**

An SAE is any untoward medical occurrence at any dose that results in death, is life-threatening (immediate risk of death), requires inpatient hospitalization or prolongation of existing hospitalization, results in persistent or significant disability/incapacity (substantial disruption of the ability to conduct normal life functions), and results in congenital anomaly/birth defect.

**Severity assessment**

The severity of adverse events will be graded according to the Common Terminology Criteria for Adverse Effects (CTCAE) V.5.

**Causality assessment**

The relationship to study drug of each adverse event will be assessed using the following definitions:

DEFINITE: distinct temporal relationship with drug treatment. Known reaction to agent or chemical group or predicted by known pharmacology. Event cannot be explained by subject’s clinical state or other factors.

PROBABLE: reasonable temporal relationship with drug treatment. Likely to be known reaction to agent or chemical group or predicted by known pharmacology. Event cannot easily be explained by subject’s clinical state or other factors.

POSSIBLE: reasonable temporal relationship with drug treatment. Event could be explained by subject’s clinical state or other factors.

UNLIKELY: poor temporal relationship with drug treatment. Event easily explained by subject’s clinical state or other factors.

UNRELATED: the event occurs prior to dosing. Event or intercurrent illness is due wholly to factors other than drug treatment.

**Reporting requirements**

Each AE will be assessed to determine if it meets the criteria for SAEs. If an SAE occurs, expedited reporting will follow IRB, local, and international regulations, as appropriate. All SAEs will be reported to IDI Scientific Review Committee, the IRB, the Uganda National Council of Science and Technology (UNCST), and the Uganda National Drug Authority (NDA). All SAEs will be reported to the sponsor within 24 h of becoming aware of the event and to the ethics committee, UNCST, and NDA within 7 days of knowing about the event. Further relevant follow-up information will be given when available and follow-up will continue until the event resolves.

All AEs will be tabulated and reported to the IRB in annual study reports. AEs and SAEs will be reported using concise medical terminology.

#### Frequency and plans for auditing trial conduct {23}

Periodic monitoring will be conducted independent of the investigators by internal monitors at the Infectious Diseases Institute. The frequency and procedures for monitoring the trial are outlined in a monitoring plan.

#### Plans for communicating important protocol amendments to relevant parties (e.g., trial participants, ethical committees) {25}

Protocol deviations will be communicated to the appropriate authorities within seven days. Amendments to the protocol will be implemented following local ethics and regulatory approvals and updated in ClinicalTrials.org.

#### Dissemination plans {31a}

Information will be developed in modalities appropriate to the target audience. Input from Community Advisory Boards (CAB) will be sought from study set-up to dissemination. Through regular meetings between CAB and the study team, key updates on study progress, new evidence external to the study, and study results will be communicated and presented in a format which is accessible to members of the community. Other communication activities may include a trial website, study Twitter account, and newsletters.

Data will be shared with consortium partners and other research groups according to data transfer agreements, with all originating RemTLAR data remaining the property of the sponsor. Access to study data will be granted to qualified individuals for research and educational purposes after approval by the relevant regulatory bodies.

A whole or part of this study results will be communicated, orally presented, and/or published in appropriate scientific journals and research forums including the IDI Research Forum and Journal Club. In addition, participants wanting to see the results of the trial can request a copy of the article from the investigators once it has been published. Full anonymity of participant’s details will be maintained throughout.

## Discussion

This is the first study that will evaluate the nature and magnitude of potential drug-drug interactions (DDI) between tenofovir/lamivudine or atazanavir/ritonavir with remdesivir. Additionally, it will characterize pharmacokinetics after a single-dose remdesivir in healthy volunteers from a typical African population in whom clinical use is anticipated. Results will inform guidance on starting doses of remdesivir in patients concurrently using tenofovir/lamivudine and atazanavir/ritonavir. Furthermore, this study will deliver pharmacokinetic datasets for remdesivir drug concentrations and demographic characteristics which could support pharmacometric approaches for simulation of new remdesivir treatment regimens.

### Trial status

Screening of participants into the study with protocol Version 3.0, September 21, 2020, began on March 19, 2021, enrolled 28 participants, and completed participant follow-up in July 2021.

## Abbreviations

AE: Adverse events
ART: Antiretroviral therapy
AUC: Area under the concentration-time curve
AUCinf: AUC from time zero to infinity
AUClast: The AUC from time zero to the last measurable concentration sampling time
Cmax: Maximum concentration
COVID-19: Coronavirus disease 2019
CRF: Case report form
CYP: Cytochrome P450
DDI: Drug-drug interaction
DOT: Directly Observed Therapy
EAU: Emergency Authorization Use
EBOV: Ebola virus
ECG: Electrocardiogram
EOS: End of study
EVD: Ebola virus disease
HIV: Human immunodeficiency virus
HPLC: High performance liquid chromatography
IDI: Infectious diseases institute
IRB: Institutional Review Board
NDA: National Drug Authority
NRTI: Nucleoside reverse transcriptase inhibitor
NNRTI: Non-nucleoside reverse transcriptase inhibitor
PK: Pharmacokinetic
SAE: Serious adverse event
TDM: Therapeutic drug monitoring
Tmax: The time to reach maximum (peak) plasma drug concentration after single-dose administration
WHO: World Health Organization
